# Trends of Patients’ Preferences in the Management of First and Early Second Trimester Pregnancy Loss Towards the Choice of Medical or Surgical Termination of Pregnancy in a Tertiary Care Center in Oman

**Published:** 2019

**Authors:** Tamima Al-Dughaishi, Mussab Mubarak Hamed Al-Jabri, Amjad Hamed Al-Haddabi, Vaidyanathan Gowri

**Affiliations:** - Sultan Qaboos University and Hospital, Muscat, Oman

**Keywords:** Abortion, Miscarriage, Misoprostol, Parity, Trends

## Abstract

**Background:**

This study aimed to find out the patients preference towards the management of pregnancy loss to either Misoprostol or dilatation and evacuation or curettage (D&C) in two different study periods.

**Methods:**

Retrospective chart review study included a total of 411 patients. The first study period was January to December 2010 and the second study period was January to December 2014. All patients were managed in Sultan Qaboos University Hospital of Oman.

**Results:**

Misoprostol was more preferable than dilatation and curettage D&C in both 2010 and 2014, with percentages of 79.30% and 69.57%, respectively. There was a slight increase in the preference toward D&C in 2014. There was a slight increase in the preference toward D&C with older age group as well as in patients with history of miscarriages.

**Conclusion:**

In comparison between 2010 and 2014 data, there was no significant change in patients’ preference. There was a slight increase in the preference toward D&C in the older age group and in patients with previous history of abortion in both years.

## Introduction

Miscarriage is defined according to World Health Organization (WHO) as “the premature loss of a fetus up to 23 weeks of pregnancy and weighing up to 500 *g*”. It accounts for about 20% of total pregnancies and 80% out of them occur in the first 12 weeks of gestation (1). The actual prevalence of miscarriage is much higher because some patients have very early miscarriages even before they know that they are pregnant. A study was conducted and patients’ hormones were evaluated every day in order to detect miscarriages as early as possible and it was found that the rate of miscarriage was 31% (2).

The patients have three choices for the management of the problem; The patients have three choices of management of miscarriage and they are expectant, medical and surgical management. In expectant management also called “Conservative management”, patient decides to wait until the miscarriage is completed naturally and the conception products are evacuated passively. It is usually the favored choice when the uterus is small or when there is few remaining products (3).

Medical management is done by Misoprostol alone, which is a prostaglandin E1 analogue, and preferred over earlier medications like methotrexate and mifepristone (4). This drug is listed in the World Health Organization (WHO) model list of essential medicine in April 2013 as a drug used for management of incomplete miscarriage as well as for prevention of postpartum hemorrhage (WHO, 2013) (5). The effect of Misoprostol depends on the dose and the route of administration (6, 7).

Kong et al. compared medical, surgical, and expectant management and they found equal efficacy. Regarding satisfaction of patients toward the type of managements, the same study suggested both surgical and medical evacuations were well accepted by the patients (8).

Shokry et al. concluded that although vaginal and surgical evacuation was more effective than Misoprostol in solving the problem, still medical treatment is effective and acceptable especially when surgical management is not available or risky or patients refuse to undergo surgical management (9).

In a study conducted by Alexandra et al. about the patients’ attitudes toward the surgical versus expectant management, it was shown that majority of the participants in the study prefer the expectant management over the surgical management (10).

Besides the one by Alexandra et al., there are not many published reports in this subject. Though the Misoprostol has been applied for more than a decade, it was available for use in our institute only from 2010. The route of administration was vaginal in most women and rectal if there was some vaginal bleeding. The dosage varied from 400 *μgr* to 800 *μgr* depending on the gestation and previous cesarean scars. The dosage and route was standardized after 2010, as it was widely advised in many guidelines including FIGO and the Royal college of Obstetricians and Gynecologists. Hence, two timelines were chosen. Before 2010, there was no standard protocol for these patients and 2014 following standardization of Misoprostol administration.

## Methods

This was a retrospective descriptive study conducted at Sultan Qaboos University Hospital (SQUH). Patients included were admitted in Gynecology ward for management of first and early second trimester miscarriage–missed miscarriage or incomplete miscarriage in the period between January to December 2010 and January to December 2014. All patients who were suitable for either medical or surgical management were included in the study. Those women who needed urgent evacuation and curettage were excluded. They were counselled on both methods and they chose either one of them. Those who chose expectant management were excluded from the study.

Ethical approval was obtained from Medical Research and Ethics Committee of SQUH. Information was retrieved on maternal characteristics such as age, gravidity, parity, and abortion. Index pregnancy details also were obtained like gestational age which was measured by ultrasound, weeks of gestation by last menstrual period (LMP), and uterine size. Statistical analysis was done on coded data using Statistical Package for the Social Sciences (SPSS) software. Data were categorized and analyzed using frequencies tables, pie charts and the comparisons were carried out using the Pearson Chi-square test.

## Results

**Figure 1.**
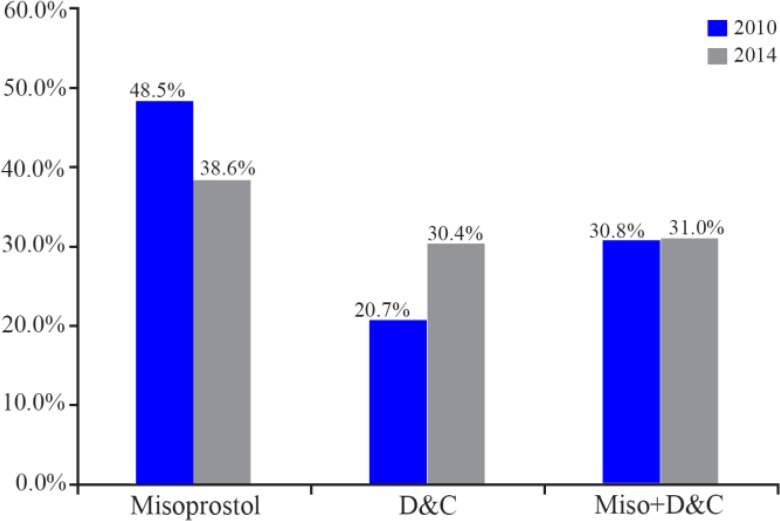
Type of pregnancy termination management in 2010 and 2014 using Misorprostol or D&C

A total of 411 patients were included in this study. All these women were admitted for management of incomplete or missed miscarriage. There were 227 patients in 2010 and 184 patients in 2014. In 2010, Misoprostol was preferred by 180 (79.30%) women while only 47 (20.70%) women preferred D&C as a primary management for their miscarriage. In 2014, Misoprostol was preferred by 128 (69.57%) women and D&C was preferred by 56 (30.43%) women (Figure 1).

The mean age of the patients included was 31.9 years in both 2010 and 2014 with minimum of 20 years in both periods and maximum of 47 years and 49 years in 2010 and 2014.

Patients were subdivided into three categories regarding their age. First group included those patients who were less than or equal to 25 years, and the second group included those patients in the age range of 26–35 and the third group included patients older than 35. In 2010, among 41 patients of first age group 35 (85.4%) chose to get Misoprostol and 6 (14.6%) preferred D&C. The second age group contained 113 patients and among them 90 patients (79.6%) preferred Misoprostol while 23 patients (20.4%) preferred D&C. The third age group included 73 patients and among them 55 patients (75.4%) preferred to take Misoprostol and 18 patients (24.6%) preferred D&C. There was no significant relationship between age group and patient’s preference as the p value of relation was >0.05. The same pattern was observed in 2014 (Table 1).

**Table 1.** Age of women and preference for misoprostol and D&C

	**2010**		**2014**	

**Age group (years)**	**Misoprostol**	**D&C**	**Misoprostol**	**D&C**
**≤25**	85.4%	14.6%	75.3%	26.7%
**26–35**	79.7%	20.3%	70.1%	29.9%
**>35**	75.3%	24.7%	66.7%	33.3%

Patients were also divided regarding their parity history into patients with no previous parity and patients having previous parity. In 2010, of the 54 nulliparous women, 44 patients (87%) started with Misoprostol and 7 patients (13%) preferred D&C. While the total of 173 patients included in the group of patients with previous parity, 133 patients (76.8%) started Misoprostol as management and 40 patients (23.2%) preferred D&C. There was a significant relation between the parity history and the patients preferences (p<0.05) (Table 2).

**Table 2.** Parity and preference for Misoprostol *vs*. D&C

	**2010**		**2014**	

	**Misoprostol**	**D&C**	**Misoprostol**	**D&C**
**Nulliparous**	87%	13%	72%	28%
**Parous**	76.8%	23.2%	68.4%	31.6%
		p<0.05		p>0.05

**Figure 2.**
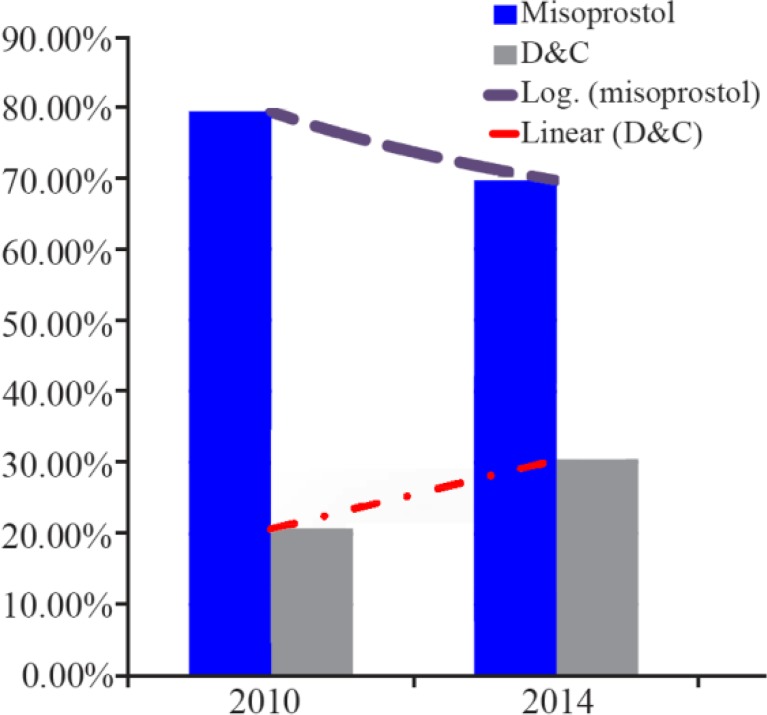
Trends of preference to Misoprostol and D&C in 2010 and 2014

In 2014, among the total of 133 patients in the parous group, 91 patients (68.4%) started with Misoprostol as management of preference and 42 patients (31.6%) preferred D&C. Of the 50 nulliparous women, 36 patients (72%) preferred Misoprostol and 14 patients (28%) underwent D&C. There was no significant relation in 2014, between parity history and patients’ preference (p>0.05). The trend was more towards dilatation and curettage and that is shown in figure 2.

Mean parity and abortions of the patients were not much different in these two times period (Table 3).

**Table 3.** Mean parity and abortions in the study group

	**2010**			**2014**		

	**Minimum**	**Maximum**	**Mean**	**Minimum**	**Maximum**	**Mean**
**Parity**	0	12	2.72	0	12	2.49
**Abortion**	0	5	0.67	0	7	0.88
**Weeks of gestation**	6	22	11.56	6	22	11.57
**Number of doses**	0	5	1.35	0	3	0.95

## Discussion

More recently, medical treatment with Misoprostol has been introduced as a cost-effective non-surgical alternative of pregnancy termination (4, 6, 7). Treatment with Misoprostol leads to an incomplete evacuation of the uterus in 20–50% of treated women according to some authors (10–13). In a study by Lemmers et al. in 2016, of the 256 participating women, only 59 (23%) accepted randomization for expectant versus curettage. That illustrates the presence of strong treatment preferences in this clinical situation (14).

In recent years, discrete-choice experiments (DCEs) have become the approach for studying patient preferences in health care. The method involves asking individuals to indicate their preference in hypothetical alternative scenarios by offering a series of choice sets from which they are to choose their preferred ones (15). However, other than a few studies, there are hardly any data on literature on patient preference.

Our study was retrospective and patients were suitable for either medical or surgical management. In comparison between 2010 and 2014 data, there was no significant change in patients’ preference (p>0.05). There was slight increase in the preference toward D&C in older age group. This slight increase in older age group could be due to their experiences with various types of managements. On the other hand, data showed that younger patients preferred Misoprostol as a management of choice in both years.

There was also a slight increase in the preference toward D&C in patients with previous history of abortion in both years. Those slight increases might be due to bad experience with Misoprostol as a management for their previous miscarriages or due to unpleasant side effect that they experienced. These are hypothetical as they were not included in our study.

Since this was a retrospective study, such choice experiments were not conducted and some conclusions were made hypothetically regarding their preferences. However, this may help the health authorities in equipping the patients with the medications or counselling the patients.

## Conclusion

The various options of miscarriage management are medical, expectant and surgical. Misoprostol is the most commonly used medication for medical management. In our institute the dosage and route of administration was standardized after 2010. When we compared the trend between 2010 and 2014, we noticed a trend of many patients towards a surgical evacuation than medical in 2014.
